# 复合层析柱净化-气相色谱-负化学源质谱法测定海洋沉积物中多溴联苯醚

**DOI:** 10.3724/SP.J.1123.2022.10006

**Published:** 2023-06-08

**Authors:** Jingyan DONG, Suping SONG, Xiumei SUN, Yanjian JIN, Qing HAO, Jian ZHU, Tiejun LI

**Affiliations:** 1.浙江海洋大学海洋与渔业研究所, 浙江 舟山 316021; 1. Marine and Fisheries Research Institute of Zhejiang Ocean University, Zhoushan 316021, China; 2.浙江省海洋水产研究所, 浙江省海洋渔业资源可持续利用技术研究重点实验室, 浙江 舟山 316021; 2. Zhejiang Marine Fisheries Research Institute, Key Laboratory of Sustainable Utilization of Technology Research for Fisheries Resources of Zhejiang Province, Zhoushan 316021, China

**Keywords:** 气相色谱-负化学源质谱, 多溴联苯醚, 沉积物, 复合层析柱净化, gas chromatography-negative chemical ionization-mass spectrometry (GC-NCI/MS), polybrominated diphenyl ethers (PBDEs), sediment, composite chromatography column purification

## Abstract

采用去活硅胶-酸化硅胶-去活硅胶-弗罗里硅土-无水硫酸钠填装的复合层析柱,结合气相色谱-负化学源质谱法(GC-NCI/MS)对海洋沉积物样品中的13种多溴联苯醚(PBDEs)进行分析检测。样品用正己烷-二氯甲烷(3∶1, v/v)混合溶剂进行超声提取,提取液经复合层析柱净化及正己烷-二氯甲烷(3∶1, v/v)混合溶剂洗脱后,利用GC-NCI/MS的选择离子监测(SIM)模式分析测定13种PBDEs。对比分析了复合层析柱中不同填料、不同洗脱剂以及不同洗脱体积对PBDEs净化效果的影响,并对GC-NCI/MS分析条件进行优化处理。最终13种PBDEs在0.1~20 μg/L内线性关系良好,相关系数(*r*^2^)>0.995(BED-209, *r*^2^>0.99),定量限(*S/N*=10)为0.002~0.126 μg/kg。以海洋沉积物样品为基质,13种PBDEs在0.2、1.0、4.0 μg/kg 3个加标水平下的平均回收率分别为85.3%~101.3%、84.8%~113.6%、86.3%~94.7%,相对标准偏差分别为4.4%~14.0%、0.4%~4.9%、1.9%~6.6%。结果表明,该方法的灵敏度和准确度高,精密度良好。应用本方法对所采集的海洋沉积物样品进行分析检测,结果显示13种PBDEs均有不同程度的检出,其中BDE-209含量较高,最高达60.49 μg/kg。实际样品检测结果表明本方法适用于海洋沉积物样品中PBDEs的准确定性定量分析。

## Abstract (en)

Polybrominated diphenyl ethers (PBDEs) are used as additive flame retardants. Because they lack the ability to form chemical bonds, PBDEs can easily enter the sediment environment. The accurate qualitative and quantitative analysis of PBDEs in sediments is of great importance for the accurate assessment of PBDE pollution in this environment. Sediments contain many impurities. Therefore, PBDEs in sediment should be purified before analysis to reduce the matrix effect. A method based on gas chromatography-negative chemical ionization-mass spectrometry (GC-NCI/MS) was developed to determine 13 PBDEs in marine sediment samples using a column packed with deactivated silica gel, acidified silica gel, Florisil, and anhydrous sodium sulfate. Sediment samples were extracted by ultrasonication with a mixed solvent of *n*-hexane-dichloromethane (3∶1, v/v). After two cycles of ultrasonic extraction, the extract was purified by a composite chromatographic column and eluted with *n*-hexane-dichloromethane (3∶1, v/v). Thirteen PBDEs were determined by GC-NCI/MS in selected-ion monitoring (SIM) mode. The effects of different fillers, eluents, and elution volumes on the purification of PBDEs in the composite column were compared and analyzed, and the GC-NCI/MS analysis conditions were optimized. Three different packing columns were used to purify the sample extract. The first column was packed with 3 g of deactivated silica, 6 g of acidic silica, 3 g of deactivated silica, 3 g of Florisil, and 6 g of anhydrous sodium sulfate; the second column was packed with 3 g of Florisil, 3 g of deactivated silica, 6 g of acidic silica, 3 g of deactivated silica, and 6 g of anhydrous sodium sulfate; and the third column was packed with 3 g of deactivated silica, 6 g of acidified silica, 3 g of deactivated silica, and 6 g of anhydrous sodium sulfate. Among these columns, that packed with 3 g of deactivated silica, 6 g of acidic silica, 3 g of deactivated silica, 3 g of Florisil, and 6 g of anhydrous sodium sulfate showed the best purification effect. The 13 PBDEs showed good linearity in the mass concentration range of 0.1-20 μg/L with correlation coefficients (*r*^2^) greater than 0.995 (decabromodiphenyl oxide (BDE-209), *r*^2^>0.99). The limits of quantification (*S/N*=10) was 0.002-0.126 μg/kg. The average recoveries of the 13 PBDEs at three spiked levels of 0.2, 1.0, and 4.0 μg/kg were 85.3%-101.3%, 84.8%-113.6%, and 86.3%-94.7% with relative standard deviations of 4.4%-14.0%, 0.4%-4.9%, and 1.9%-6.6%, respectively. These findings indicate that the method has high sensitivity and accuracy as well as good precision. Finally, the method was applied to the analysis and detection of PBDEs in actual marine sediment samples. The results revealed that the sediment samples contained different contents of the 13 PBDEs, and high detection rates were obtained for lower-brominated PBDE homologs. The detection rate of bis(4-bromophenyl) ether (BDE-15) was 100%, and the detected content of BDE-209 was as high as 60.49 μg/kg. These results demonstrate that the developed method is suitable for the accurate qualitative and quantitative analysis of PBDEs in marine sediment samples.

多溴联苯醚(polybrominated diphenyl ethers, PBDEs)是溴系阻燃剂中的一类阻燃物质,由于其具有较高的阻燃效率和良好的耐热性能,被广泛应用于家电、纺织品、塑料制品等各种商品中,是诸多工业产品中不可或缺的成分^[1]^。因缺乏化学键的束缚,PBDEs极易逸散到大气中,并通过干湿沉降的作用进入到水体、土壤和生物体内,又因其具有较强的脂溶性、持久性、高毒性,而极易随食物链在生物体内富集,并易对生态环境和人类健康构成较大威胁^[2,3]^。研究表明,PBDEs已广泛存在于各种环境介质和生物体中,如大气^[4]^、土壤^[5]^、水体^[6]^、沉积物^[7]^、海产品^[8]^和人体^[9]^。2009年5月,《斯德哥尔摩公约》第四次缔约方大会已将五溴联苯醚和八溴联苯醚列为新型持久性有机污染物^[10]^。目前PBDEs占阻燃剂总量的75%以上,工业用途以五溴、八溴和十溴联苯醚最为常见^[11]^。

PBDEs在进入周围环境后会通过大气传输或地表径流进入到附近海洋环境中,并在海洋沉积物中蓄积^[12]^。目前关于PBDEs的提取方法主要有加速溶剂萃取^[13]^、超声提取^[14]^、索氏提取^[15]^等,净化方法主要有固相萃取^[16]^、凝胶渗透色谱^[17]^、复合层析柱^[18]^等,检测方法主要有气相色谱-质谱法(GC-MS)^[19]^、气相色谱-串联质谱法(GC-MS/MS)^[20]^、液相色谱-串联质谱法(LC-MS/MS)等^[21]^。沉积物中所含杂质较多,基质较为复杂,易受环境干扰,因此为了减少基质效应,一般在对沉积物中的PBDEs分析时先进行净化处理。本研究拟采用超声提取-复合层析柱净化结合气相色谱-负化学源质谱法(GC-NCI/MS)对沉积物中的PBDEs进行检测,以正己烷-二氯甲烷(3∶1, v/v)为萃取溶剂,采用超声提取法提取沉积物中的PBDEs,再用复合层析柱对提取液进行净化处理,最后使用GC-NCI/MS进行测定。此种检测方法净化效果好,灵敏度和准确性高,回收率较高,可用于对实际样品中PBDEs的检测。

## 1 实验部分

### 1.1 仪器、试剂与材料

7890B-7000C气相色谱-质谱联用仪(美国Agilent公司); R210旋转蒸发仪(瑞士Buchi公司); Centrifuge5810高速离心机(德国Eppendorf公司); N-EVAP-112氮吹仪(美国Organomation公司);SK8200GT超声波清洗器(上海科导超声仪器有限公司); MS3旋涡混合器(德国IKA公司)。

正己烷、二氯甲烷、甲苯、异辛烷、丙酮(农残级,上海安谱实验科技股份有限公司);浓硫酸、浓盐酸(优级纯,国药集团化学试剂有限公司);无水硫酸钠(农残级,上海麦克林生化科技有限公司); 50~70目铜粉、100~200目弗罗里硅土、60~200目硅胶(上海安谱科学仪器有限公司);内径17 mm具砂板闪式层析柱(北京欣维尔玻璃仪器有限公司)。

13种PBDEs混合标准溶液(溶剂为甲苯),包括4-溴二苯醚(BDE-3, CAS号:101-55-3, 50.5 mg/L)、4,4'-二溴二苯醚(BDE-15, CAS号:2050-47-7, 50.2 mg/L)、2',3,4-三溴二苯醚(BDE-25, CAS号:147217-77-4, 50.0 mg/L)、2,4,4'-三溴二苯醚(BDE-28, CAS号:41318-75-6, 50.0 mg/L)、2,2',4,4'-四溴二苯醚(BDE-47, CAS号:5436-43-1, 50.0 mg/L)、2,2',4,4',5-五溴二苯醚(BDE-99, CAS号:60348-60-9, 50.1 mg/L)、2,2',4,4',6-五溴二苯醚(BDE-100, CAS号:189084-64-8, 50.1 mg/L)、2,2',4,4',5,5'-六溴二苯醚(BDE-153, CAS号:68631-49-2, 50.0 mg/L)、2,2',4,4',5,6'-六溴二苯醚(BDE-154, CAS号:207122-15-4, 50.1 mg/L)、2,2',3,4,4',5',6-七溴二苯醚(BDE-183, CAS号:207122-16-5, 50.1 mg/L)、2,2',3,4,4',5,5',6-八溴二苯醚(BDE-203, CAS号:337513-72-1, 50.0 mg/L)、2,2',3,3',4,4',5,5',6-九溴二苯醚(BDE-206, CAS号:63387-28-0, 50.0 mg/L)、十溴二苯醚(BDE-209, CAS号:1163-19-5, 50.0 mg/L),购自上海安谱科学仪器有限公司。将13种PBDEs混合标准溶液用异辛烷逐级稀释至10、5、1 mg/L,配制成标准使用液,于-20 ℃冰箱内保存,保质期为3个月。

### 1.2 样品前处理

#### 1.2.1 样品的制备和前处理材料的预处理

沉积物样品采集自浙江省乐清市乐清湾,于4 ℃以下冷藏、密封、避光保存。将沉积物样品冷冻干燥48 h,挑拣出碎石杂质,研磨均匀,过80目筛,备用。

复合硅胶层析柱的制备:取60~200目的硅胶,在150 ℃下烘干2 h制成活化硅胶,干燥冷却后放置在螺旋瓶盖玻璃瓶中备用;称取100 g活化后的硅胶,加入10 g的水降活,搅拌均匀制成去活硅胶,放置过夜后使用;再称取100 g的活化硅胶,逐滴加入44 g浓硫酸,搅拌至无块状物制成酸化硅胶;取一定量100~200目的弗罗里硅土,于150 ℃下烘干2 h,干燥保存。于内径为17 mm的层析柱中,依次装入3 g去活硅胶、6 g酸化珪胶、3 g去活硅胶、3 g弗罗里硅土、6 g无水硫酸钠,湿法装柱。

铜粉制备:铜粉用浓盐酸-水(1∶1, v/v)浸泡10 min,倾去酸,用水洗至中性,再用丙酮洗涤3次,最后置于盛有丙酮的具塞瓶中,使用时用氮气吹干。

#### 1.2.2 提取

称取沉积物样品5 g(干重, 精确到0.01 g),置于50 mL离心管中,先后加入7.5 mL正己烷和2.5 mL二氯甲烷,涡旋振荡5 min使其完全混匀,然后控制水浴温度在25 ℃以下,超声萃取25 min,再以6000 r/min离心5 min,提取上清液至另一离心管中。再按上述方法重复提取一次,合并提取液,加入制好的铜粉,超声萃取25 min,静置2 h,再以6000 r/min离心5 min,将上清液提取至浓缩瓶中,控制水浴40 ℃以下,将提取液旋蒸至约1 mL,待净化。

#### 1.2.3 净化

用60 mL正己烷-二氯甲烷(4∶1, v/v)混合溶剂预淋洗复合层析净化柱,将浓缩液用15 mL正己烷分3次复溶并加入到净化柱中,再用60 mL正己烷-二氯甲烷(3∶1, v/v)混合溶剂对净化柱进行洗脱,收集流出液于浓缩瓶中,旋蒸至近干,再准确加入1 mL异辛烷复溶,转移至气相小瓶内,待GC-NCI/MS检测。

### 1.3 仪器条件

色谱柱:DB-5 ht色谱柱(15 m×0.25 mm×0.1 μm);色谱柱升温程序:起始温度110 ℃,保持1 min,以30 ℃/min升温至330 ℃,保持8 min;进样口温度:290 ℃;载气:高纯氦气;流速:梯度流量模式,初始值1.2 mL/min,保持10 min,以32 mL/min的速率上升至3 mL/min;进样方式:脉冲不分流;进样体积:2 μL。

质谱离子源为负化学离子源,电离能量235 eV,离子源温度280 ℃,传输线温度300 ℃,溶剂延迟时间3 min。扫描方式为选择离子监测(SIM)模式,定性、定量离子参见表1。

**表1:** 多溴联苯醚的质谱参数

Compound	Retention time/min	Quantitative ion (m/z)	Qualitative ion (m/z)
4-Bromophenoxybenzene (BDE-3)	3.678	79.3	81.3
Bis(4-bromophenyl) ether (BDE-15)	4.755	79.3	81.3
2,3',4-Tribromodiphenyl ether (BDE-25)	5.404	161.1	79.3
2,4,4'- Tribronmodiphenyl ether (BDE-28)	5.497	161.1	79.3
2,2',4,4'-Tetrabromodiphenyl ether (BDE-47)	6.187	161.1	81.3
2,2',4,4',5-Pentabromodiphenyl ether (BDE-99)	6.683	161.1	81.3
2,2',4,4',6-Pentabromodiphenyl ether (BDE-100)	6.817	161.1	81.3
2,2',4,4',5,5'-Hexabromodiphenyl ether (BDE-153)	7.206	81.3	79.3
2,2',4,4', 5,6'- Hexabromodiphenyl ether (BDE-154)	7.389	81.3	79.3
2,2',3,4,4',5,6-Heptabromodiphenyl ether (BDE-183)	7.947	161.1	81.3
2,2',3,4,4',5,5',6'-Octabromodiphenyl ether (BDE-203)	8.673	79.3	81.3
Nonabromodiphenyl ether (BDE-206)	9.709	486.9	81.3
Decabromodiphenyl oxide (BDE-209)	10.576	488.9	486.9

## 2 结果与讨论

### 2.1 样品前处理优化

#### 2.1.1 填料的优化

沉积物样品基质较为复杂,含有腐殖质、硫化物、色素以及各种污染物。如净化效果不佳会导致目标化合物在保留时间上出现偏差或PBDEs出现分解,分离度差,直接对后续GC-NCI/MS检测产生不良影响,不仅会对仪器设备造成损害而且会影响检测结果,因此有效去除样品中的杂质对优化净化方法显得尤为重要。

采用复合层析柱对样品提取液进行净化处理,层析柱填料选用去活硅胶-酸化硅胶-去活硅胶-弗罗里硅土-无水硫酸钠(自下而上,湿装法),去活硅胶是由活化硅胶加入一定比例的水制备而成,形成硅醇基可对样品中的杂质进行吸附。酸化硅胶用于去除样品中的脂肪、硫化物、色素等杂质,无水硫酸钠主要用来去除提取液中的水分。弗罗里硅土是硅胶键合氧化镁,主要由SiO_2_、MgO和Na_2_SO_4_ 3种成分组成,多孔,具有较大比表面积,极性较强,适合从非极性样品基质中分离低极性目标化合物,如多氯联苯(PCBs)、PBDEs等,具有重现性好、回收率高的优点,常用于对土壤、动植物组织、植物样品的提取液进行净化处理。因此选择在复合硅胶柱上层加一层弗罗里硅土填料对层析柱进行优化,优化后的复合层析柱能有效提高样品在气相色谱中的响应值。

称取5 g沉积物样品(干重)并向样品中加入5 μg/L的13种PBDEs混合标准溶液1 mL,在提取方式保持不变的条件下,分别采用3种不同填料的层析柱对样品提取液进行净化,PBEDs各组分回收率如图1所示。由图1可以看出,使用弗罗里硅土装填在上层的1号层析柱(从底部依次装填3 g去活硅胶-6 g酸化硅胶-3 g去活硅胶-3 g弗罗里硅土-6 g无水硫酸钠)得到的加标回收率最高,回收率为80%~115%,且较为稳定,相对标准偏差(RSD)为0.4%~4.9%,平行性好;而在下层装填弗罗里硅土的2号层析柱(从底部依次装填3 g弗罗里硅土-3 g去活硅胶-6 g酸化硅胶-3 g去活硅胶-6 g无水硫酸钠)则显示其加标回收率结果最差,回收率为66%~120%,偏差也较大,RSD为3.6%~30.4%;不填充弗罗里硅土的3号酸性硅胶层析柱(从底部依次装填3 g去活硅胶-6 g酸化硅胶-3 g去活硅胶-6 g无水硫酸钠)显示的结果稳定性差,偏差大,回收率为68%~119%, RSD为9.9%~16.8%。这一结果表明弗罗里硅土装填在上层的复合层析柱效果要优于另外两种复合层析柱,从理论推断这可能是由于硅胶作为吸附剂具有一定的极性,对目标化合物的作用是氢键或偶极相互作用,而PBDEs属于弱极性化合物,与硅胶的作用力较弱,同时吸附杂质的能力也较弱,杂质与目标化合物不能分开,在洗脱剂的作用下容易造成共流出,导致实验结果重复性差,偏差较大。但当弗罗里硅土在上层时便可以优先有效分离提取液中的脂类杂质,再经由酸化硅胶段对残留的色素、脂类、硫化物进行分离再净化,因此所得回收率高,重复性好,偏差小;而当弗罗里硅土在下层时提取液先流经酸化硅胶段,而酸化硅胶属于破坏性净化方式,是使用浓硫酸达到去除油脂、色素和硫化物的目的,这一方式会引起化合物的不稳定,在杂质较多时可能导致目标化合物与杂质一同被吸附,从而导致回收率的降低。实验表明,1号层析柱的回收率更高,偏差更小,最终选择此种填料层析柱作为PBDEs的提取净化柱。

**图1:**
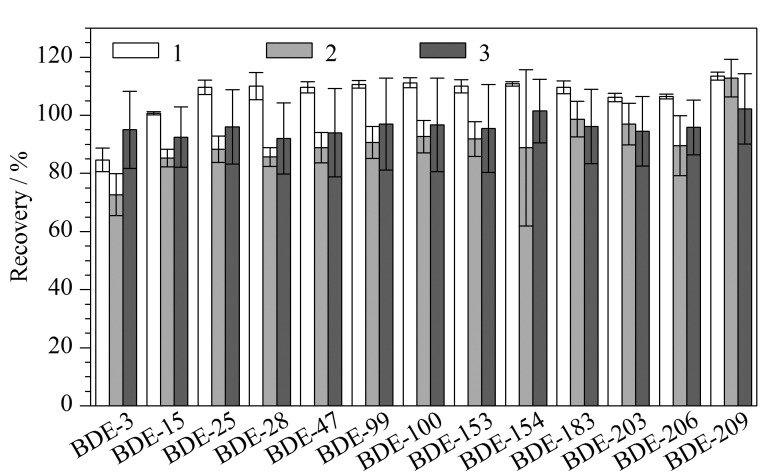
不同复合层析柱对多溴联苯醚回收率的影响(*n*=6)

#### 2.1.2 洗脱溶剂的选择

实验过程中发现洗脱剂的选择对样品回收率有很大影响,正己烷是PBDEs在净化过程中常用的洗脱溶剂,从图2中可以看出,选用正己烷作洗脱溶剂时洗脱效果不佳,回收率偏低,回收率为53%~109%。当选用正己烷-二氯甲烷(3∶1, v/v)为洗脱溶剂时,回收率良好,各化合物的回收率较均衡(80%~115%),RSD为0.4%~4.9%。综合考虑,本实验选用正己烷-二氯甲烷(3∶1, v/v)混合溶剂作为洗脱溶剂。

**图2:**
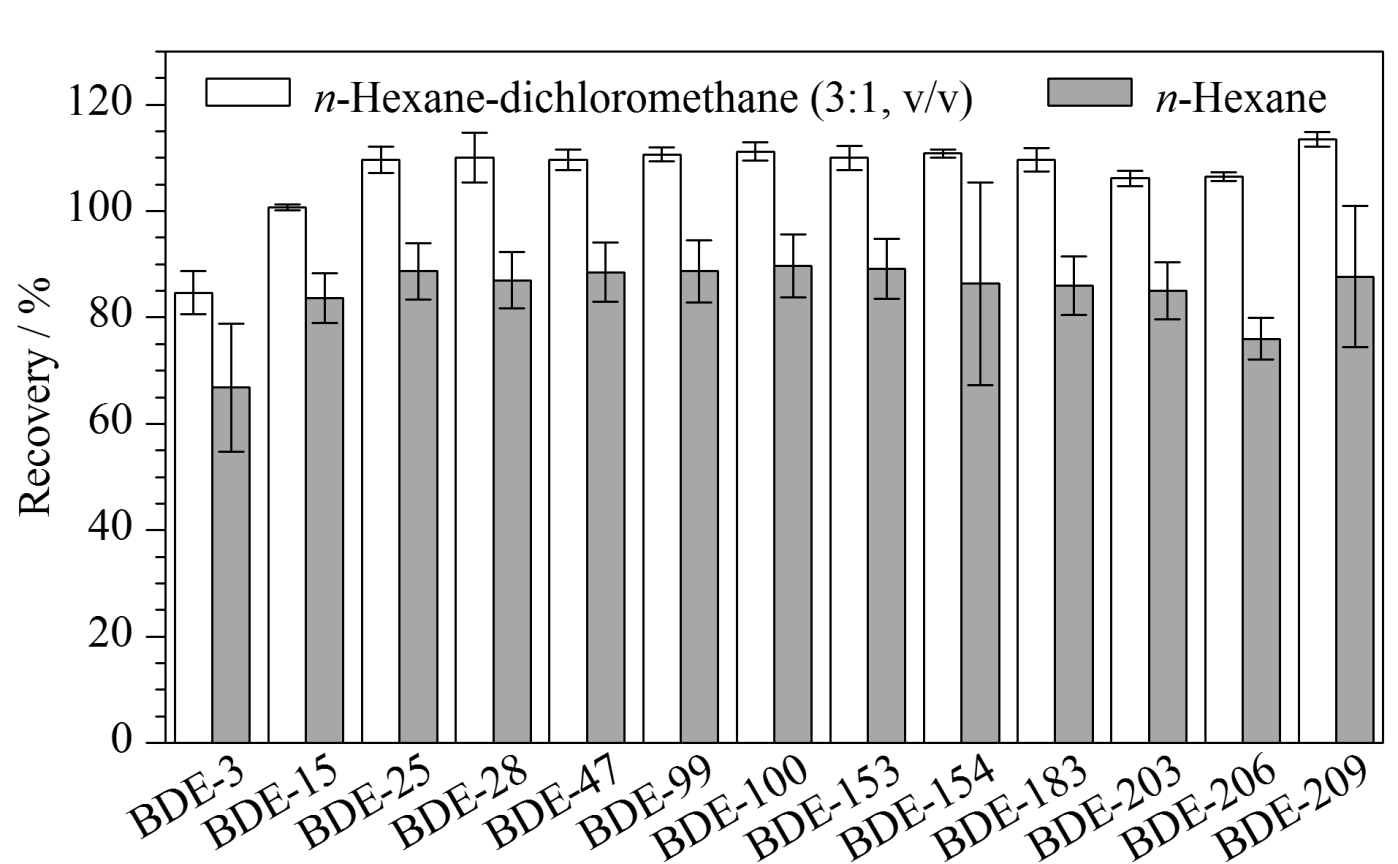
不同洗脱溶剂对多溴联苯醚回收率的影响(*n*=6)

#### 2.1.3 洗脱体积的选择

采用分段收集洗脱液的方法来对洗脱溶剂的体积进行选择,在样品过复合层析柱后共使用60 mL的正己烷-二氯甲烷(3∶1,v/v)混合溶液进行洗脱,分段收集,分别收集30 mL、15 mL、15 mL的洗脱液,依次将其编号为A、B、C,测定洗脱液中的目标化合物,计算出每段洗脱的绝对回收率占3次洗脱总回收率的比率。由表2可以看出,A段洗脱过程中大部分目标化合物已伴随洗脱液流出,B、C段洗脱液中仅含有少量目标化合物,且三段洗脱液中目标化合物总回收率为80%~115%(图2),综合考虑最终选用60 mL洗脱液对复合层析柱进行洗脱。

**表2:** 3段洗脱液中的PBDEs回收率占总回收率的权重

Compound	A	B	C
BDE-3	78	10	12
BDE-15	90	7	3
BDE-25	96	3	1
BDE-28	94	4	2
BDE-47	94	4	1
BDE-99	97	3	1
BDE-100	95	4	1
BDE-153	98	2	0
BDE-154	91	5	4
BDE-183	93	5	2
BDE-203	90	6	4
BDE-206	84	9	8
BDE-209	81	10	9

A, B, C: eluates orderly eluted with 30, 15, 15 mL *n*-hexane-dichloromethane (3∶1, v/v).

### 2.2 GC-NCI/MS条件的优化

由于BDE-209的相对分子质量较大,所取代的溴原子个数较多,在高温条件下性质不稳定,易在色谱柱上分解,为使BDE-209尽早出峰,避免保留时间过长而分解,因此选用15 m的短柱用于PBDEs的测定。升温程序采用起始温度110 ℃,保持1 min,以30 ℃/min升温至330 ℃,保持8 min,进样口温度设置为290 ℃,能够使BDE-209充分气化。PBDEs类化合物在分子结构上一般都带有强电负性原子或基团,选用GC-NCI/MS检测相对来说可以获得较高的灵敏度,13种PBDEs的保留时间和特征离子的选择见表1。在对仪器条件进行优化时,为准确定量分析,试用十氯联苯醚(PCB-209,CAS号:2051-24-3,纯度96.16%,英国LGC公司)作内标物质,但因各组分PBDEs的溴原子取代数量不同,质谱分析时其与PCB-209的响应因子会有差异,定量分析时发现低溴代BDEs的测定结果被低估,因此在方法评价及实际样品检测中采用了外标法定量。图3为13种PBDEs与内标PCB-209混合标准溶液的色谱图,从图中可以看出峰形较好,各组分能够有效分离,方法的选择性良好。

**图3:**
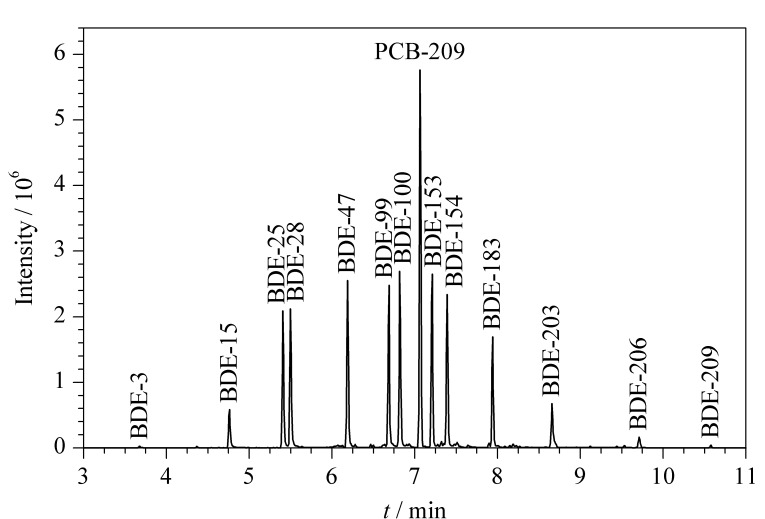
13种多溴联苯醚的色谱图

### 2.3 线性关系和检出限

将13种PBDEs混合标准溶液用异辛烷逐级稀释,配制20、10、5、1、0.5、0.1 μg/L的系列标准溶液,从低质量浓度到高质量浓度依次进样,采用本方法进行测定,以目标组分的质量浓度为横坐标,以响应面积为纵坐标进行线性拟合,绘制标准曲线,外标法定量。结果显示,13种PBDEs在0.1~20 μg/L内,除BDE-209外,相关系数(*r*^2^)均大于0.995,线性关系良好。为避免沉积物本底干扰,选用石英砂为空白样品进行检出限及定量限试验。称取5 g石英砂,向石英砂中添加1.0 ng PBDEs标准物质,按照1.2节进行前处理及分析,以3倍信噪比(*S/N*=3)相对应的浓度确定该方法PBDEs的检出限,以10倍信噪比(*S/N*=10)所对应的浓度确定该方法PBDEs的定量限,结果显示13种PBDEs的检出限为0.001~0.038 μg/kg,定量限为0.002~0.126 μg/kg,说明该方法具有较高的灵敏度。13种PBDEs的相关系数、检出限和定量限见表3。

**表3:** 13种多溴联苯醚的相关系数、检出限和定量限

No.	Compound	r^2^	LOD/(μg/kg)	LOQ/(μg/kg)
1	BDE-3	0.9999	0.034	0.113
2	BDE-15	0.9998	0.002	0.007
3	BDE-25	0.9999	0.001	0.002
4	BDE-28	0.9999	0.001	0.002
5	BDE-47	0.9999	0.001	0.002
6	BDE-99	0.9999	0.001	0.002
7	BDE-100	0.9999	0.001	0.002
8	BDE-153	0.9997	0.001	0.002
9	BDE-154	0.9997	0.001	0.002
10	BDE-183	0.9999	0.001	0.003
11	BDE-203	0.9997	0.002	0.006
12	BDE-206	0.9957	0.008	0.028
13	BDE-209	0.9901	0.038	0.126

### 2.4 准确度和精密度

按优化后的实验方法对沉积物样品进行检测,在沉积物样品中加入13种PBDEs混合标准溶液,进行准确度和精密度试验,试验结果扣除样品本底值。试验的准确度用加标回收率表示,精密度用RSD表示。称取5 g沉积物样品(干重),同时进行3个水平(0.2、1.0、4.0 μg/kg)的加标回收试验,每个加标水平做6次平行测定。如表4所示,所测平均回收率为84.8%~113.6%, RSD为0.4%~14.0%。结果表明该方法的回收率高,精密度好。

**表4:** 沉积物中13种目标化合物的加标回收率及相对标准偏差(*n*=6)

No.	Compound	0.2 μg/kg			1.0 μg/kg			4.0 μg/kg		
		Recovery/%	RSD/%		Recovery/%	RSD/%		Recovery/%		RSD/%
1	BDE-3	101.3	4.4		84.8	4.9		89.4	4.1	
2	BDE-15	97.4	10.5		100.7	0.4		87.8	5.2	
3	BDE-25	90.4	12.3		109.7	2.2		86.3	6.3	
4	BDE-28	85.3	11.6		109.9	4.2		87.6	4.6	
5	BDE-47	88.3	14.0		109.6	1.6		87.0	6.4	
6	BDE-99	89.1	12.5		110.8	1.0		86.3	5.8	
7	BDE-100	90.4	9.5		111.1	1.5		89.9	6.4	
8	BDE-153	91.6	10.9		110.1	2.0		88.3	6.1	
9	BDE-154	95.8	9.6		111.0	0.8		93.4	6.6	
10	BDE-183	95.6	11.5		109.6	1.9		91.4	6.6	
11	BDE-203	90.8	9.3		106.1	1.5		89.8	5.6	
12	BDE-206	98.8	9.9		106.5	0.8		92.6	3.2	
13	BDE-209	97.5	11.6		113.6	1.1		94.7	1.9	

### 2.5 实际样品检测

将建立好的检测方法应用在实际沉积物样品检测中,沉积物样品在2021年4~5月完成采集,采样时以晴天为主,采集的样品为乐清湾的表层沉积物,根据监测规范布设26个站位点,检测结果见表5。从所检测的26个沉积物样品结果中可以看出,13种PBDEs均有不同程度的检出。BDE-209检出含量较高,最高达60.49 μg/kg,明显高于其他PBDEs,其余组分的检测结果显示污染程度较轻,这一现象与其他学者对沉积物中PBDEs的分析结果一致^[22⇓-24]^, 即PBDEs各组分含量以BDE-209为主。从含量方面看,BDE-209是所有同系物中最为主要的同系物,其主要来源可能是工业品中使用的PBDEs,低溴代联苯醚主要可能来自于高溴代联苯醚,特别是BDE-209的脱溴降解或长期累积作用而来,而BDE-209含量较高与目前十溴联苯醚是作为主要溴系阻燃剂产品使用有关。从实际沉积物样品中各类PBDEs的检出率来看,低溴代同系物检出率较高,其中BDE-15检出率为100%。

**表5:** 沉积物样品中多溴联苯醚的含量

Site	BDE-3	BDE-15	BDE-25	BDE-28	BDE-47	BDE-99	BDE-100	BDE-153	BDE-154	BDE-183	BDE-203	BDE-206	BDE-209
1	0.95	0.28	0.08	-	0.46	0.45	0.93	0.10	0.29	0.08	-	0.64	60.49
2	0.21	0.24	0.07	-	0.06	0.07	0.04	0.04	0.06	-	0.08	0.25	23.19
3	0.75	0.28	-	-	0.08	0.11	0.06	0.04	0.08	0.05	0.08	0.22	18.06
4	-	0.22	0.05	-	0.03	0.07	0.09	0.04	0.07	0.11	0.07	0.05	5.51
5	0.57	0.22	0.04	-	0.03	-	-	0.04	0.06	0.07	0.07	0.03	4.60
6	0.40	0.22	0.03	-	0.03	-	-	0.03	0.07	0.05	-	-	2.56
7	2.22	0.12	0.02	0.02	0.02	-	0.15	0.03	0.01	0.04	-	-	3.65
8	2.08	0.12	0.03	-	0.02	0.06	0.13	0.03	0.01	0.05	0.06	-	3.75
9	1.65	0.05	0.03	-	0.02	0.06	0.14	0.03	-	-	-	-	2.06
10	1.38	0.17	0.03	-	0.02	-	0.15	0.03	-	0.04	-	0.12	2.64
11	15.13	0.05	0.02	-	0.03	-	-	0.02	-	0.05	0.08	0.11	17.74
12	1.38	0.07	0.03	-	0.02	-	0.13	0.03	-	-	0.05	-	2.30
13	8.82	0.07	0.03	0.02	0.02	-	-	0.02	-	0.07	0.06	0.07	11.88
14	0.10	0.03	0.02	-	0.02	0.03	-	0.02	0.07	0.01	0.05	-	-
15	0.51	0.04	0.02	-	0.02	0.04	0.08	0.02	0.07	0.03	0.05	0.01	2.54
16	-	0.03	-	-	0.02	0.04	0.02	0.02	0.07	-	0.05	-	0.43
17	0.06	0.04	0.02	0.02	0.02	0.04	0.03	-	0.08	0.03	0.08	0.21	10.77
18	0.62	0.04	0.02	0.02	0.02	0.04	-	-	0.07	0.03	0.06	0.03	4.25
19	0.60	0.03	-	-	-	0.02	-	-	0.07	0.01	-	-	-
20	0.71	0.05	-	-	0.01	0.02	-	-	0.07	0.01	-	-	-
21	0.42	0.07	0.01	0.01	0.01	0.04	0.03	0.01	0.07	0.02	-	-	0.68
22	0.14	0.04	0.02	0.02	-	-	-	0.03	0.08	0.04	-	-	3.28
23	0.61	0.04	0.02	-	0.01	-	0.11	0.02	0.06	0.03	0.04	-	1.96
24	0.35	0.05	0.02	-	0.01	0.02	-	-	0.06	0.02	0.04	-	-
25	1.74	0.04	0.02	-	0.01	0.03	0.09	0.02	0.09	0.05	-	0.08	10.20
26	0.42	0.05	0.02	-	0.02	0.04	0.12	0.02	0.06	0.03	0.05	-	1.16

-: not detected.

## 3 结论

本研究建立了13种PBDEs的复合层析柱净化-气相色谱-负化学源质谱检测方法,对填料的选择、洗脱溶剂种类及洗脱体积等前处理条件进行优化,在优化的GC-NCI/MS测试条件下,13种PBDEs在较短时间内完全分离,且绝大部分目标化合物响应较好。方法验证结果表明,方法的检出限、准确度和精密度可满足沉积物样品中PBDEs分析检测的要求。将建立的方法应用于浙江省乐清湾沉积物样品检测,结果显示乐清湾沉积物中均有不同程度的PBDEs残留,表明本方法可大规模应用于沉积物实际样品的分析。因本文采样数量有限,不足以评价乐清湾海域PBDEs的污染现状,可采用该方法进一步开展详细研究。

## References

[1] Torre A, Navarro I, Sanz P, et al. J Hazard Mater, 2020, 382: 121009 31454611 10.1016/j.jhazmat.2019.121009

[2] Guo N N, Meng S L, Chen J Z. Chinese Agricultural Science Bulletin, 2019, 35(25): 159

[3] Liang X, Zhu S, Chen P, et al. Environ Pollut, 2010, 158(7): 2387 20483516 10.1016/j.envpol.2010.04.008

[4] Chakraborty P, Zhang G, Li J, et al. Sci Total Environ, 2019, 649: 1653 30172482 10.1016/j.scitotenv.2018.07.414

[5] Wang S, Zhang S, Huang H, et al. Environ Pollut, 2014, 184: 405 24113474 10.1016/j.envpol.2013.09.021

[6] Trinh M M, Tsai C L, Chang M B. Sci Total Environ, 2019, 649: 388 30176451 10.1016/j.scitotenv.2018.08.204

[7] Yang Y L, Pan J, Li Y, et al. Chinese Science Bulletin, 2003, 48(21): 2244

[8] Lu L, Mi Y, Sun F L, et al. Chinese Journal of Health Laboratory Technology, 2022, 32(5): 517

[9] Dimitriadou L, Malarvannan G, Covaci A, et al. Sci Total Environ, 2016, 539: 350 26367190 10.1016/j.scitotenv.2015.08.137

[10] Zheng X B, Luo X J, Zeng Y H, et al. Environ Sci Technol, 2015, 49(2): 785 25525742 10.1021/es503748w

[11] Jia Y J. [MS Dissertation]. Beijing: Beijing University of Chemical Technology, 2011

[12] Li Y Y. [PhD Dissertation]. Shanghai: Fudan University, 2014

[13] Fang L P, Zhao Y X, Lu B W, et al. Physical Testing and Chemical Analysis (Part B: Chemical Analysis), 2018, 54(7): 825

[14] He X F, Xiong J, Wang M C, et al. Advanced Measurement and Laboratory Management, 2014, 54(7): 3

[15] Zhang Z Y, Yu X Q, Wang X G. Chemical Enterprise Management, 2020(24): 47

[16] Jin J, Song S J, Peng Z J, et al. Chinese Journal of Analytical Chemistry, 2021, 49(4): 520

[17] Sun Y M, Xu M, Li G Y, et al. Chemistry, 2015, 78(2): 170

[18] Ma S H, Rao Q X, Zhang Q C, et al. Acta Agriculturae Universitatis Jiangxiensis, 2020, 42(5): 1050

[19] Xun Z B, Wang X Y, Lü D Z. Chinese Journal of Tropical Agriculture, 2021, 41(4): 82

[20] Li F, Peng W C, Liang L. Plastics Science and Technology, 2022, 50(3): 79

[21] Xu N B, Qian F Z, Feng J Y, et al. Chinese Journal of Analytical Chemistry, 2015, 43(2): 251

[22] Chen L. [MS Dissertation]. Shanghai: Shanghai University, 2015

[23] Chen X P, Peng B Q, Lü S P, et al. Environmental Science, 2016, 37(5): 1771

[24] Zhou P, Lin K F, Yu H J, et al. China Environmental Science, 2016, 36(1): 149

